# Acupuncture versus sham acupuncture and usual care for Antiandrogen-Induced hot fLashes in prostate cancer (AVAIL): study protocol for a randomized clinical trial

**DOI:** 10.1186/s12906-023-04218-y

**Published:** 2023-10-27

**Authors:** Zongshi Qin, Zhiwei Zang, Jianyong Yu, Jianqin Lv, Ning Li, Jialing Zhang, Mingxiao Yang, Joey S. W. Kwong, Ran Pang, Jianfeng Wang, Zhengyu Cui, Yongpei Yu, Haibo Wang, Yidan Zhu, Yifang Yuan, Xiao Li, Yangfeng Wu, Jiani Wu

**Affiliations:** 1 School of Public Health, Peking University, Beijing, China; 2 Peking University Clinical Research Institutes, Beijing, China; 3 Department of Acupuncture, Yantai Hospital of Traditional Chinese Medicine, Yantai, China; 4 Department of Urology, Yantai Hospital of Traditional Chinese Medicine, Yantai, China; 5 Department of Integrative Medicine, West China Hospital, Sichuan University, Chengdu, China; 6 School of Chinese Medicine, Hong Kong Chinese Medicine Clinical Study Centre, Hong Kong Baptist University, Hong Kong, Hong Kong SAR, China; 7 Department of Medicine, Integrative Medicine Service, Memorial Sloan Kettering Cancer Center, New York City, NY USA; 8 Department of Health Policy, Department of Clinical Epidemiology, National Center for Child Health and Development, Tokyo, Japan; 9 Department of Urology, Guang’anmen Hospital, China Academy of Chinese Medical Sciences, Beijing, China; 10 Department of Urology, China-Japan Friendship Hospital, Beijing, China; 11 Department of Chinese Medicine, Shanghai East Hospital, Tongji University, Shanghai, China; 12 Department of Urology, Jiangsu Cancer Hospital & Jiangsu Institute of Cancer Research & Nanjing Medical University Affiliated Cancer Hospital, Nanjing, China; 13 Department of Acupuncture, Guang’anmen Hospital, China Academy of Chinese Medical Sciences, Beijing, China

**Keywords:** Acupuncture, Sham acupuncture, Quality of life, Prostate cancer, Hot flashes, Randomized controlled trial, Study protocol

## Abstract

**Background:**

Hot flashes are the common and debilitating symptom among prostate cancer (PCa) patients undergoing androgen deprivation therapy (ADT). Strong evidence from multiple rigorously designed studies indicated that pharmacological option such as venlafaxine provides partial relief, but the tolerability is poor when dose is not tapered. Hence, alternative therapy is needed. Previous studies reported that acupuncture may be helpful in the management of hot flashes. However, the insufficient randomized controlled trial limited the quality of evidence.

**Methods:**

Five hospitals will recruit 120 acupuncture naïve patients with moderate-to-severe hot flashes after prostate cancer received ADT in China from February 2023 to December 2024. Participants will be randomly 2:1:1 allocated to the 18 sessions of verum acupuncture at true acupuncture points plus usual care, 18 sessions of non-penetrating sham acupuncture at non-acupuncture points plus usual care, or usual care alone over 6 weeks. The primary outcome measure is the change of mean weekly hot flashes symptom severity score (HFSSS) at the end of treatment compared with baseline.

**Expected Results and Conclusion:**

We will be able to measure the effectiveness of acupuncture for patients with PCa suffering from ADT-induced hot flashes and whether acupuncture is superior to sham acupuncture and usual care. The proposed acupuncture treatment might provide an alternative option for those patients.

**Trial Registration:**

Clinicaltrials.gov (NCT05069467).

**Supplementary Information:**

The online version contains supplementary material available at 10.1186/s12906-023-04218-y.

## Introduction

Prostate cancer is a common form of cancer and accounts for 29% of cancer diagnoses in males [1]. With the development of diagnostic and therapeutic strategies, survival periods of prostate cancer patients have significantly increased. Androgen deprivation therapy (ADT) is a primary treatment option for prostate cancer and has been demonstrated to improve overall survival when used with radiation for intermediate- and high-risk localized disease, as well as locally advanced and node-positive disease, and after surgery for node-positive disease [2]. However, the use of ADT is associated with testosterone depletion, leading to several side effects such as hot flashes and loss of bone density to mood swings, weight gain, and erectile dysfunction among nearly all men [3]. Of these side effects, hot flashes have been reported as the most troublesome symptom by 30.7% of PCa patients undergoing ADT [4]. Patients often experience sudden and transient episodes of heat and sweating, with possible co-occurring palpitations and anxiety, which usually persist long-term [5, 6]. Although hot flashes are not considered a fatal morbidity, they may interfere with adherence to lifesaving therapies or ablative therapies that are used to prevent or treat cancer [7, 8].

Currently, the majority of studies for hot flashes have focused on evaluating treatments in breast cancer patients or postmenopausal women [3]. Venlafaxine, a selective serotonin reuptake inhibitor, has demonstrated efficacy in both breast cancer women and prostate cancer men with hot flashes. It has been recommended for clinical practice in men with strong evidence from multiple rigorously designed studies [9]. However, the use of venlafaxine can be limited in some prostate cancer patients due to the occurrence of side effects such as nausea, headache, dry mouth, dizziness, insomnia, and constipation [10]. Acupuncture was initially reported to be a promising therapy for ADT-induced hot flashes in a case series study [11]. This was followed by several case series studies, again reporting promising results [12, 13]. Only one pilot randomized controlled trial compared acupuncture to electro-acupuncture has been conducted in the past few decades, without a sham control, and draw similar conclusions [14]. Although acupuncture is a nonpharmacologic therapy and has been confirmed as helpful in the management of hot flashes among breast cancer survivors, [15] currently there is no robust evidence demonstrating its efficacy in men [14, 16]. With a placebo effect influencing up to 30% of patients, the efficacy of acupuncture needs to be confirmed in prospective randomized controlled trials with a sham-controlled design [14, 17]

Therefore, a few clinical questions remain unclear: (1) whether acupuncture is more effective than usual care (2)? whether verum acupuncture is more effective than sham acupuncture and, if so, what is the effect size? To answer these questions, we designed this Acupuncture versus sham acupuncture and usual care for Antiandrogen-Induced hot fLashes in prostate cancer (AVAIL) study, the first multi-center, large sample size randomized controlled trial to evaluate the efficacy and safety of acupuncture for hot flashes among PCa patients.

## Patients and methods

### The AVAIL study design

The AVAIL is a multicenter, sham-controlled, three-arm, randomized clinical trial. This study has been registered on clinicaltrials.gov (identifier: NCT05069467). Ethical approval has been obtained from the Research Ethics Board of the Guang’anmen Hospital, China Academy of Chinese Medical Sciences. Local institutional approval has been obtained in each of the five sub-centers. The institutional review boards at the local sites approved the protocol, and all patients will be approved to join this study until provide written informed consent. The AVAIL is conducted at five clinical centers including the West China Hospital, Yantai Hospital of Traditional Chinese Medicine, Jiangsu Cancer Hospital & Jiangsu Institute of Cancer Research & Nanjing Medical University Affiliated Cancer Hospital, East Hospital affiliated to Tongji University, and Guang’anmen Hospital affiliated to China Academy of Chinese Medicine. The enrolment started on June 2020 but was interrupted because of the coronavirus disease 2019 pandemic until 1 December 2022. During 2020 to 2022, the screening and consultant is active and ongoing, but no patients were included. The last site (East Hospital affiliated to Tongji University) is activated in May 2023. Five hospitals will recruit 120 acupuncture naïve patients with moderate-to-severe hot flashes after prostate cancer received ADT in China from February 2023 to December 2024. Participants will be randomly 2:1:1 allocated to the 18 sessions of verum acupuncture at true acupuncture points plus usual care, 18 sessions of non-penetrating sham acupuncture at non-acupuncture points plus usual care, or usual care alone over 6 weeks. The table S1 summarize the items for Standard Protocol Items for Randomized Trials (SPIRIT).

### Patients

Urologists, oncologists, and acupuncturists at each participating center will screen patients for eligibility. The inclusion and exclusion criteria are presented in Table 1. All patients participating in the study should provide a written informed consent form prior to any study procedures. At the initial visit, the research assistants will collect demographic information, medical history, blood work results, and details about the participant’s previous and current treatment. Patients will be asked to complete a daily hot flashes diary during the screening period (baseline week). Additionally, the Functional Assessment of Cancer Therapy-Prostate (FACT-P), The International Index of Erectile Function (IIEF-5), the Zung Self-Rating Anxiety Scale (SAS), and the Zung Self-Rating Depression Scale (SDS) will also be collected during this period.

**Table 1:** Inclusion and exclusion criteria

**Inclusion criteria** 1. Men aging from 18 to 75 years with histologically confirmed primary invasive carcinoma of the prostate 2. Receiving a stable dose of hormonal therapy for at least 4 weeks before the study with plans to continue for at least an additional 6 weeks after participation to the AVAIL study 3. With at least 14 or more times hot flashes per week during screening 4. Patients did not receive acupuncture before 5. Patients have an estimated survival time of at least 1 year
**Exclusion criteria** 1. The patient did not recover from side-effects of the surgery on the prostate 2. Patients with cognitive dysfunction who cannot cooperate with treatment and assessment 3. Use of drugs that may affect hot flashes symptoms: androgens, estrogens, progestational agents, gabapentin, pregabalin within the past 3 months. Antidepressants will be allowed if the patient had been on a stable dose for at least 1 month and did not plan to modify this treatment during the ensuing 14 weeks.

### Verum acupuncture

Licensed acupuncturists with more than 5 years of experience were responsible for administering interventions three times per week for 6 weeks. The points include bilateral Xinshu (BL15), Shenshu (BL23), Zhongliao (BL33), Sanyinjiao (SP6), and Yinlingquan (SP9). The location and insertion depth of these points and procedure was described in eTable S1 and S2. The acupuncture needles (30 or 40 mm and 0.25 mm gauge; Soochow, Hwato) will be inserted and manipulated until De Qi, a sensation of soreness and tingling. During a 30-minutes duration, manual manipulation of each needle lasted 10 s and repeated a total of 3 times with an interval of 10 min. The acupuncturists were instructed no to discuss treatment effects with the patients.

### Sham acupuncture

This protocol was designed to provide a sham control. The procedure of sham acupuncture will be the same for the sham acupuncture, except for the following: the acupuncturist selected the same number of non-acupuncture points. To maintain blinding in patients, the acupuncture ritual is the same in both manual and sham acupuncture groups. Instead of eliciting De Qi, the Streitberger needles will be minimally manipulated to avoid eliciting sensations other than initial contact with skin. After sterilization, the acupuncturists will place a small plastic ring over the acupuncture point/non-acupuncture point and fix the ring with plaster, and then inserted the needle through the plaster inside the ring.

### Usual care

The patients in the usual care group will receive acupuncture treatment same as the verum acupuncture for free after a waiting period of 14 weeks and upon completing the entire visit. Usual care for these patients will comprise clinical interviews, counseling, and health education. This will include diary-keeping to help identify and avoid triggers for hot flashes, maintaining a cool environment during the day and while sleeping, and avoid excessive consumption of caffeine and alcohol, as well as refraining from smoking.

### Allocation

**Fig. 1:**
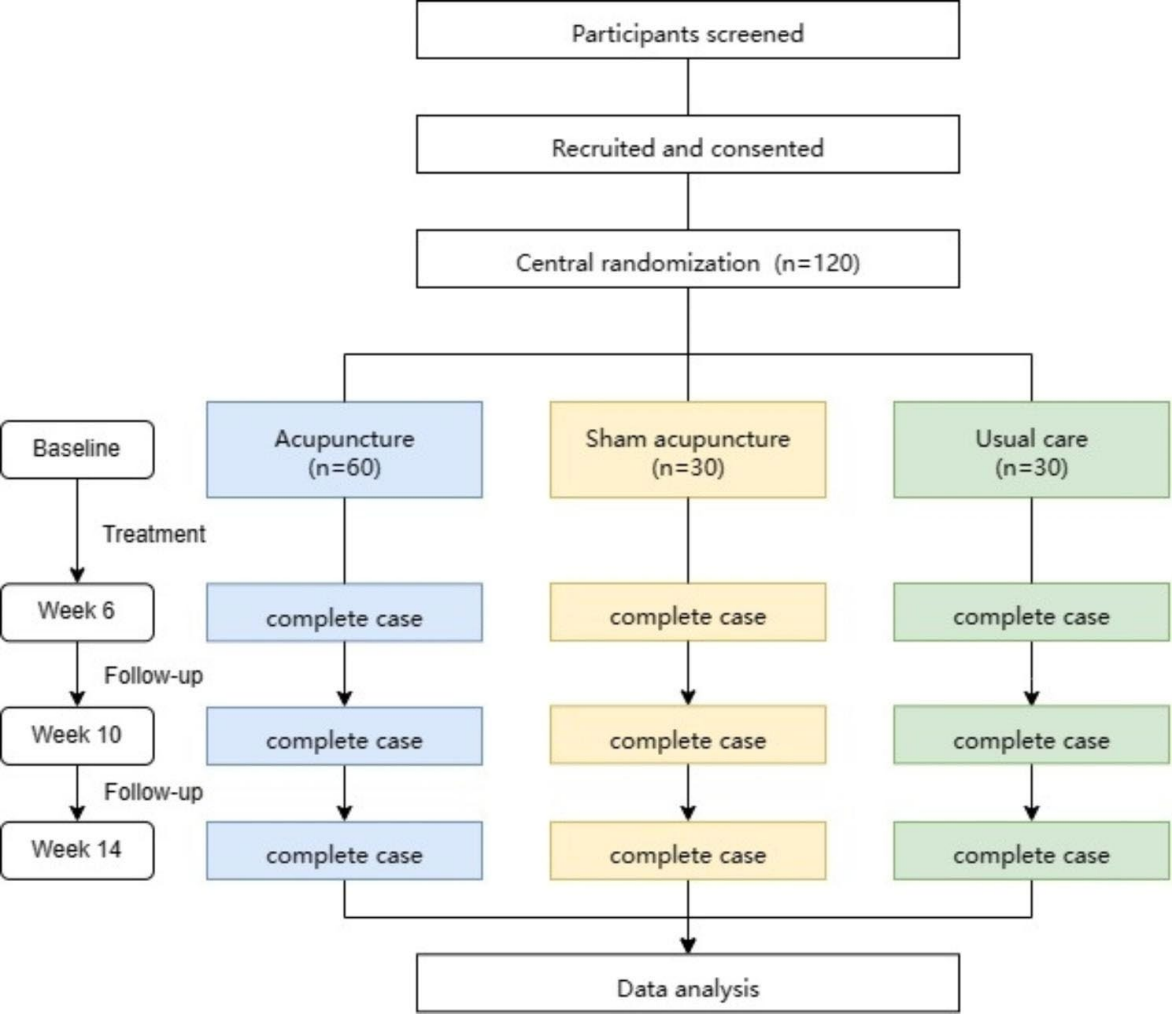
Flow chart of AVAIL trial

**Fig. 2:**
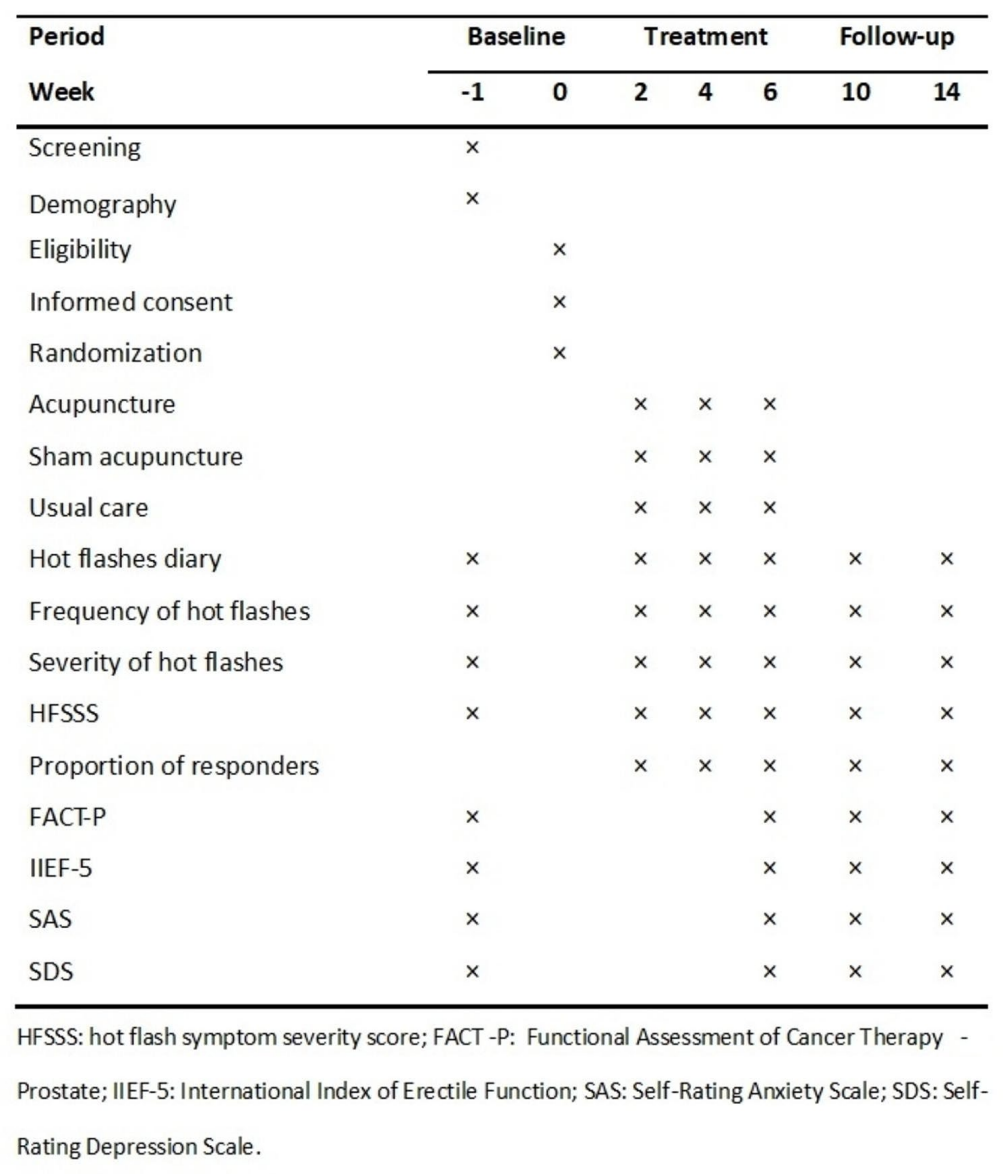
Schedule of activities during the AVAIL trial for patients

Participants were randomly allocated in a ratio of 2:1:1 into one of the three groups: (1) verum acupuncture, (2) sham acupuncture, or (3) usual care group by an interactive online computer program. The randomization scheme for the study was produced by SAS version 9.4 software (SAS Institute, Inc). Figures 1 and 2 represent the study flow chart and the schedule of activities during the AVAIL trial for patients, respectively.

### Masking and blinding assessment

The principal investigator, study investigators, patients, study staff, and the statistician will be blinded to the treatment assignments among interventions. The acupuncturists cannot be blinded to verum acupuncture or the sham owing to the characteristics of acupuncture procedure. Patients allocated to both verum acupuncture and sham acupuncture groups will be asked to guess which type of acupuncture (verum acupuncture, sham acupuncture, or unclear) they had received after the end of last treatment [18]. Bang’s blinding index will be estimated to evaluate successiveness of blinding [19].

### Outcomes

The primary outcome is weekly mean hot flash symptom severity score (HFSSS), [20] which is a validated symptom-measuring tool used to assess the frequency and severity grade of hot flashes. The severity of hot flashes is assessed based on the duration of hot flashes and physical and emotional symptoms, it is scored as mild (grade 1, less than 3 min, light physical symptoms, no emotional symptoms and no action needed), moderate (grade 2, up to 5 min, moderate physical symptoms, mild anxiety, some irritability and loss of concentration, need to use a fan, loosen clothing, and remove bedding), severe (grade 3, up to 10 min, physical symptoms described as feeling hotter or very hot, heavy perspiration, dizziness, nausea, shortness of breath, weakness, and extreme discomfort; moderate anxiety and irritability; need to loosen clothing, change clothing, and change bedding), and very severe (grade 4, up to 30 min, very significant physical and emotional symptoms and the need to change clothing, bedding, towel off, take a shower, and take rest). Patients will be given the questionnaire and taught how to complete it. Patients will be asked to make a daily record of the number of hot flashes and corresponding severity. The weekly mean HFSSS represented the mean value of these scores during the week preceding the visit. Secondary outcomes related to HFSSS include response rate, the responder will be defined according to a 50% reduction of weekly mean HFSSS compared with baseline; the frequency of hot flashes at each visit; the severity of hot flashes at each visit. Other secondary outcomes including FACT-P (Functional Assessment of Cancer Therapy-Prostate). It is a questionnaire used to assess the health-related quality of life in men with prostate cancer [21]. The questionnaire consists of 39 items and is a relevant tool worldwide. It includes the FACT-G (general) subscales, which measure general HRQoL in cancer patients, and a 12-item prostate subscale. The International Index of Erectile Function (IIEF-5) is a shortened version of the IIEF questionnaire used to evaluate erectile dysfunction [22]. It consists of five questions that assess different aspects of sexual function, including erectile and orgasmic functions. The score is calculated by summing up the ordinal responses to the five items, with higher scores indicating better sexual function. The Zung Self-Rating Anxiety Scale (SAS) consists of 20 items that assess common symptoms of anxiety, such as tension, fear, worry, and nervousness. Each item is rated on a Likert-type scale ranging from 1 to 4. Scores on the scale can range from 20 to 80, with higher scores indicating more severe symptoms of anxiety [23]. The Zung Self-Rating Depression Scale (SDS) is familiar with SAS. Scores on the SDS range from 20 to 80, with higher scores indicating more severe symptoms of depression [24]. Table 2 summarized the primary and secondary endpoints.

**Table 2:** Primary and secondary endpoints

**Primary endpoint** Change from baseline to 6 weeks for the HFSSS
**Secondary endpoints** 1. Change from baseline to 2, 4, 10, and 14 weeks for the HFSSS 2. Change from baseline to 2, 4, 6, 10, and 14 weeks for the frequency of hot flashes 3. Changes from baseline to 2, 4, 6, 10, and 14 weeks for the severity of hot flashes 4. The proportion of responders (at least a 50% reduction in the HFSSS) at 6, 10, and 14 weeks 5. Changes from baseline to 6, 10, and 14 weeks for the FACT-P 6. Changes from baseline to 6, 10, and 14 weeks for the IIEF-5 7. Changes from baseline to 6, 10, and 14 weeks for the SAS 8. Changes from baseline to 6, 10, and 14 weeks for the SDS

HFSSS: hot flash symptom severity score; FACT-P: Functional Assessment of Cancer Therapy-Prostate; IIEF-5: International Index of Erectile Function; SAS: Self-Rating Anxiety Scale; SDS: Self-Rating Depression Scale.

### Adverse events

Adverse events are defined as any new untoward medical event (symptoms, side-effect, or reaction) in a patient after interventions. This study will not be monitored by an independent data and safety monitoring committee because acupuncture or sham acupuncture is considered a minimal-risk study in numerous clinical trials and that they were not associated with any serious AEs, low frequency of low-grade AEs potentially related to acupuncture was observed during our previous studies [25, 26]. Any AE-related data will be collected by questioning the patient after each treatment or self-reported by participants within 24 h. AEs will be reported electronically within 5 calendar days. If an AE is considered as possibly related to the study, unexpected and serious, it will be reported rapidly to the Steering Committee and reported the Research Ethics Board within 48 h, or 24 h if life-threatening.

### Sample size calculation

The sample size is estimated using data from previous clinical studies [12, 14]. We assumed that patients who receive verum acupuncture, sham acupuncture, and usual care will have a 7.5-, 5-, and 3.5-point diminishment after 6 weeks of treatment. With these data, we estimated a 2.5-point difference effect between acupuncture and sham acupuncture and 4-point difference between acupuncture treatment and usual care, with assuming the standard deviation for the HFSSS reduction would be 4.6, probabilities of 5% for type I error, and 10% for type II error. To ensure adequate power for each individual hypothesis testing, a sample size of 96 will be required, which consists of 48 subjects in the verum acupuncture group, 24 subjects in the sham acupuncture group and 24 subjects in the usual care group. Considering 20% of the participants might drop out early or be affected by surgical procedure, resulting in a final estimated sample size of 60 patients in verum acupuncture group, 30 patients in sham acupuncture, and 30 patients in usual care, for a total of 120 participants. Further, we considered a difference smaller than 50% difference compared with baseline not to be clinically relevant.

### Statistical analysis

All statistical analyses will be conducted on an intent-to-treat basis, defined as all participants who have received randomization with whole baseline data. Summary tables will be provided for all variables as appropriate. For continuous variables, means and standard deviations will be presented, unless the variable has a skewed distribution, in which medians, 25th and 75th percentiles will be presented. For categorical variables, the number and percentage of participants with each category will be presented. For each variable (continuous or categorical), the missing values will be reported. We will compare clinical and demographic characteristics between groups at baseline by one-way analysis of variance (ANOVA), chi-square test or Kruskal-Wallis test as appropriate.

The continuous longitudinal data will be analyzed by mixed model for repeated measures with a covariance matrix included. To analyze the primary outcome (mean weekly changes of HFSSS at week 6 compared with baseline), a linear mixed effects model will be used to estimate the differences from baseline and between groups. The random effect for participants will be included, the fixed effects include 1 within-subject variable (time, 5 strata, 2, 4, 6, 10, and 14 weeks) and 1 between-subject variable (group, 3 strata). According to the priori specifications, contrast comparisons will be performed to assess the effects between (1) verum acupuncture versus sham acupuncture and (2) acupuncture versus usual care. The assumptions made for mixed-effect models will be check thoroughly by residual plots. Other secondary outcomes with the same pattern of longitudinal data will be analyzed use the similar way as primary analysis. Pairwise comparisons between the acupuncture group and the two other groups will be conducted only if the omnibus F-test statistics indicates that the null hypothesis should be rejected. For the primary outcomes, a two-tailed alpha level of 0.05 will be consider indicating statistical significance. We will use a fixed sequence procedure for multiple comparisons among groups thus the type I error will not be inflated (acupuncture vs. usual care and then acupuncture vs. sham acupuncture). Because of the exploratory nature of secondary analysis, the *P* values will not be adjusted for multiple comparisons.

For response rate of HFSSS, a logistic generalized linear mixed model with link function of logit for repeated measures will be used. The dependent variable will be response or non-response at each scheduled post-baseline visit. The definition of responder is a 50% decrease of HFSSS compared with baseline. The logistic generalized linear mixed model will include the baseline HFSSS as a covariate, with treatment group, visit, and treatment-by-visit interaction as fixed effects. Between-group comparisons at each visit will be estimated by differences between least-squares means from the treatment-by-visit interaction and be presented as odds ratios or ratio difference. For both continuous and categorical variables, 95% confidence intervals will be calculated as appropriate.

For missing data of primary outcome, the multiple imputation method will be used under the missing-at-random (MAR) assumption to generate 100 imputed data sets of HFSSS. To assess the robustness of the primary analyses, the sensitivity analyses will be tested with a tipping-point approach by using specified shift and scale parameters. All analyses and summaries will be performed by using SAS version 9.4.

### Study management

The study is supervised by the Executive Committee led by the principal investigators. At each centre, a site lead investigator is designated to oversee the study locally. The principal investigators, along with the site lead investigators, the biostatistician, and the project leader will constitute the Steering Committee that will provide guidance and direction for the overall study. An independent Data and Safety Monitoring Committee comprising urologist, oncologist, acupuncturist, and biostatistician is formed to assess serious AEs and provide recommendations to the study team through the Executive Committee. The China Academy of Chinese Medical Sciences centrally coordinate and internally monitor study quality.

## Discussion

Hot flashes are a pervasive and debilitating symptom among prostate cancer patients undergoing ADT, which can significantly impact their quality of life and lead to poor treatment tolerance and early discontinuation. While acupuncture has shown promising results in managing hot flashes among women with breast cancer, [27] its use among prostate cancer patients on ADT has not been extensively studied. A previous randomized controlled trial assessed the efficacy of acupuncture and gabapentin for breast cancer survivors with hot flashes. The study found that sham acupuncture worked better than gabapentin or the placebo, and both electroacupuncture and sham acupuncture gave better relief than gabapentin [15]. These findings suggest that acupuncture may be a viable alternative to medication for managing hot flashes.

Studies also reported that acupuncture may relieve hot flashes in prostate cancer patients on ADT. In a Swedish pilot study, seven men treated with acupuncture for hot flashes due to ADT for prostate cancer, six of them completed a 10-week course of acupuncture, and the frequency of hot flashes was reduced between 50 and 70% at various time points. In another study, 14 men who were experiencing hot flashes due to hormone therapy for prostate cancer received acupuncture twice a week for 30 min at a time for four weeks. Two weeks after receiving acupuncture, their discomfort from daily hot flashes had dropped more than half, and after eight months, their mean score was still low. However, none of these studies compared real acupuncture with a sham or placebo control, making it difficult to assess the efficacy of acupuncture for ADT-induced hot flashes in men with prostate cancer [28]. While previous studies have provided promising results for acupuncture as a potential treatment, none have compared real acupuncture with a sham or placebo control. This AVAIL study aims to provide robust evidence of whether acupuncture could be used for ADT-induced hot flashes of prostate cancer patients. The study has the largest sample size among randomized trials with sham acupuncture and usual care control to date. The results of the AVAIL study will help determine whether acupuncture is a viable alternative to medication for managing hot flashes in prostate cancer patients on ADT.

### Electronic supplementary material

Below is the link to the electronic supplementary material.

Supplementary Material 1

## Data Availability

Not applicable.

## Abbreviations

AVAIL: Acupuncture Versus sham acupuncture and usual care for Antiandrogen-Induced hot fLashes in prostate cancer
ADT: Androgen deprivation therapy
HFSSS: Hot flash symptom severity score
FACT-P: Functional Assessment of Cancer Therapy-Prostate
IIEF-5: International Index of Erectile Function
SAS: Self-rating anxiety scale
SDS: Self-rating depression scale
PCa: Prostate cancer
SPIRIT: Standard Protocol Items for Randomized Trials

## Declarations

### Trial status

The participants are currently being recruited for the present study.
